# Advances in Stroke Neuroprotection and Repair: Bridging Acute Interventions and Regenerative Biomarkers

**DOI:** 10.3390/biomedicines14071531

**Published:** 2026-07-08

**Authors:** Konstantinos Tsamakis

**Affiliations:** 1 Department of Psychiatry, Faculty of Medicine, University of Thessaly, 41110 Larisa, Greece; ktsamakis@uth.gr; 2 Institute of Psychiatry, Psychology and Neuroscience (IoPPN), King’s College London, London SE5 8AB, UK; 3 Department of Clinical Sciences, New Anglia University, George Hill AI-2640, Anguilla; 4 Institute of Medical, Biomedical and Allied Health Education, City St George’s, University of London, London SW17 0RE, UK

## 1. Optimizing Hyperacute Reperfusion: Expanding the Interventional Window

The foundational milestone of neuroprotection is the rapid, mechanical restoration of blood flow to ischemic brain tissue. While endovascular treatment (EVT) has established an unquestionable standard of care for severe strokes, its application in patients presenting with mild clinical deficits remains highly debated. Addressing this critical clinical dilemma, researchers in this issue evaluated the effectiveness and safety of emergency mechanical thrombectomy in large vessel occlusion (LVO) stroke patients presenting with low National Institutes of Health Stroke Scale (NIHSS) scores of 5 or less who exhibit predominant cortical signs [1].

Historically, these patients were frequently managed with conservative medical therapy due to their low initial symptom score. However, this study adds vital clarity to existing knowledge by demonstrating that isolated, major cortical signs (such as aphasia or spatial neglect) flag a massive volume of highly vulnerable, threatened brain tissue. The authors’ findings confirm that prompt endovascular intervention in this specific cohort achieves better rates of functional independence at 90 days without significantly increasing symptomatic intracranial hemorrhage rates. This demonstrates that clinical selection should rely on the anatomical footprint of the tissue-at-risk rather than arbitrary bedside impairment scores alone—a major leap forward for hyperacute neuroprotection.

## 2. Proactive Neuroprotection: Disrupting the Thromboembolic Cascade

To protect the brain over time, clinicians must also focus on preventing recurrent embolic showers that disrupt early tissue repair. This is particularly challenging in cryptogenic strokes, which are frequently driven by undetected, paroxysmal atrial fibrillation (AF). The structural design and rationale of the AVANT-GARDE trial published in this issue directly confronts this diagnostic vulnerability [2].

The trial establishes a rigorous, head-to-head electrocardiogram comparison between standard 24 h Holter monitoring and continuous, single-lead wearable devices in ischemic stroke survivors. While traditional short-term monitoring routinely misses transient, asymptomatic arrhythmic episodes, this study highlights the clinical utility of prolonged, consumer-friendly wearable technologies. By validating these devices in a structured clinical trial, the work adds a vital preventative dimension to the concept of neuroprotection. Intercepting silent AF early allows for the immediate initiation of targeted oral anticoagulation, shielding recovering neural networks from secondary thromboembolic insults.

## 3. Visualizing Neuroplasticity: Neuroimaging Biomarkers of Cortical Repair

Once the acute and secondary risks are stabilized, the clinical focus transitions entirely toward tissue repair and functional remodeling. A major hurdle in modern neurorehabilitation has been the lack of objective, real-time metrics to quantify cortical reorganization. Bridging this gap, a comprehensive systematic review and narrative discussion in this issue evaluated the role of functional neuroimaging as a biomarker for tracking upper limb recovery following non-invasive brain stimulation [3].

Non-invasive brain stimulation—such as repetitive transcranial magnetic stimulation (rTMS) or transcranial direct current stimulation (tDCS)—seeks to modulate cortical excitability to promote motor recovery. This review systematically synthesizes the existing literature to demonstrate how functional neuroimaging (including fMRI and functional near-infrared spectroscopy) acts as a direct visual biomarker of treatment efficacy. It maps changes in regional cerebral blood flow and interhemispheric connectivity, adding clear evidence that clinical motor improvements correlate precisely with physical, visible network rewiring. This shifts neurorehabilitation away from empirical “trial-and-error” approaches toward biomarker-driven, personalized restorative protocols.

## 4. Somatic and Molecular Interconnections in Neurological Recovery

The continuum of stroke neuroprotection and repair depends heavily on the interplay between acute microstructural alterations, systemic immunomolecular cascades, and downstream neurocognitive adaptation. Mapping these interactions requires robust neuroimaging and structural biomarkers capable of predicting tissue recovery and functional trajectories from the earliest stages of ischemia. In the hyperacute and acute windows, advanced imaging techniques like Diffusion Tensor Imaging (DTI) and tractography have emerged as invaluable prognostic tools, allowing clinicians to track longitudinal white matter integrity and directly evaluate a patient’s long-term recovery potential [4]. Beyond active axonal paths, pre-existing structural vulnerabilities within the cerebral architecture also dictate the brain’s resilience to injury. For instance, the burden of leukoaraiosis—assessed via standard CT or MRI visual rating scales—serves as an essential prognostic marker that reliably forecasts post-stroke functional outcomes, motor recovery capacity, early neurological deterioration, and the risk of hemorrhagic transformation [5].

Concurrently, the acute cellular response remains a major determinant of cellular survival and vascular stability within the ischemic zone. The activation of systemic immune cascades—specifically the deployment of neutrophil extracellular traps (NETs)—has surfaced as a critical pathophysiological process, offering a highly promising molecular target for refining stroke prognosis and developing novel immunomodulatory therapeutics [6]. These physical and molecular disruptions ultimately extend into long-term psychological and higher-order neurobiological deficits. The persistent neurobiological stress following a cerebrovascular event heavily alters neurochemical pathways, contributing to the high incidence of post-stroke anxiety and emotional mood disorders [7]. Together, these diverse perspectives emphasize that improving stroke outcomes requires a multi-dimensional framework that addresses everything from macrostructural neuroimaging markers to microcellular immunomodulatory signaling.

## 5. Conclusions

In conclusion, the articles compiled in this Special Issue provide a comprehensive overview of modern stroke care. By advancing our approach to mechanical reperfusion in mild-deficit LVO strokes [1], refining long-term arrhythmia surveillance through wearables [2], and validating functional neuroimaging as a guide for neuroplastic repair [3], these studies collectively redefine the boundaries of clinical recovery. We extend our sincere gratitude to the authors, reviewers, and editorial team for their contributions. These insights confirm that the future of stroke therapeutics lies in a unified approach—where hyperacute neuroprotection and long-term neuro-restoration are treated as a single, continuous effort to preserve the human mind.

## Data Availability

Not applicable.
